# UNUSUAL PHENOLIC COMPOUNDS CONTRIBUTE TO ECOPHYSIOLOGICAL PERFORMANCE IN THE PURPLE-COLORED GREEN ALGA *ZYGOGONIUM ERICETORUM* (ZYGNEMATOPHYCEAE, STREPTOPHYTA) FROM A HIGH-ALPINE HABITAT

**DOI:** 10.1111/jpy.12075

**Published:** 2013-05-23

**Authors:** Siegfried Aigner, Daniel Remias, Ulf Karsten, Andreas Holzinger

**Affiliations:** Institute of Botany, University of InnsbruckSternwartestraße 15, Innsbruck, A-6020, Austria; Institute of Pharmacy, University of InnsbruckInnrain 80-82, Innsbruck, A-6020, Austria; Institute of Biological Sciences, Applied Ecology and Phycology, University of RostockAlbert-Einstein-Straße 3, Rostock, D-18057, Germany

**Keywords:** desiccation, photoprotection, pigment, tannins, ultrastructure, UV acclimation

## Abstract

The filamentous green alga *Zygogonium ericetorum* (Zygnematophyceae, Streptophyta) was collected in a high-alpine rivulet in Tyrol, Austria. Two different morphotypes of this alga were found: a purple morph with a visible purple vacuolar content and a green morph lacking this coloration. These morphotypes were compared with respect to their secondary metabolites, ultrastructure, and ecophysiological properties. Colorimetric tests with aqueous extracts of the purple morph indicated the presence of soluble compounds such as phenolics and hydrolyzable tannins. High-performance liquid chromatography-screening showed that *Z. ericetorum* contained several large phenolic peaks with absorption maxima at ∼280 nm and sometimes with minor maxima at ∼380 nm. Such compounds are uncommon for freshwater green microalgae, and could contribute to protect the organism against increased UV and visible (VIS) irradiation. The purple *Z. ericetorum* contained larger amounts (per dry weight) of the putative phenolic substances than the green morph; exposure to irradiation may be a key factor for accumulation of these phenolic compounds. Transmission electron microscopy of the purple morph showed massive vacuolization with homogenous medium electron-dense content in the cell periphery, which possibly contains the secondary compounds. In contrast, the green morph had smaller, electron-translucent vacuoles. The ecophysiological data on photosynthesis and desiccation tolerance indicated that increasing photon fluence densities led to much higher relative electron transport rates (rETR) in the purple than in the green morph. These data suggest that the secondary metabolites in the purple morph are important for light acclimation in high-alpine habitats. However, the green morph recovered better after 4 d of rehydration following desiccation stress.

*Zygogonium ericetorum* Kützing is a filamentous zygnematophycean green alga, which occurs in extreme habitats such as open soils or acidic ponds (Lynn and Brock 1969, Hoppert et al. 2004, Kleeberg et al. 2006), waterbodies with high contents of heavy metals (Boyd et al. 2009), and high-alpine ephemeral streamlets (Holzinger et al. 2010). At high altitudes, the alga is exposed to harsh environmental conditions, including intense photosynthetically active radiation (PAR) and ultraviolet radiation (UVR), as investigated for *Z. ericetorum* by Cockell and Rothschild (1999); regular periods of desiccation (Holzinger et al. 2010); a short growing season; and sharp temperature fluctuations with occasional frost events, even during summer (for meteorological details see Karsten et al. 2010). Being subject to these different abiotic stresses, *Z. ericetorum* must possess a range of protective mechanisms to guarantee long-term survival. For example, photoprotection is needed to allow for the harmless dissipation of excess absorbed radiation energy, which otherwise would saturate photosynthetic electron transport and drive the transfer of electrons to oxygen (Mehler reaction), thereby generating reactive oxygen species (ROS; Saibo et al. 2009). Excess ROS inactivate photosystem II (PSII) reaction centers by causing chronic photoinhibition, can induce oxidation and depolymerization of nucleic acids and the breakage of peptide bonds (Kranner et al. 2010), and have other destructive intracellular effects (Hadacek et al. 2011). Rothschild (1994) showed in *Z. ericetorum* that the addition of CO_2_ enhanced carbon fixation rates during daylight hours, which led to the conclusion that the naturally occurring midday depression in photosynthesis was unlikely to be due to photoinhibition.

Currently, the phylogenetic position of the genus *Zygogonium* remains unclear, with no features that clearly separate *Zygogonium* from *Zygnema* (Hall et al. 2008, Stancheva et al. 2012). However, for this study we use the species name *Z. ericetorum*, which has marked vacuolar-confined purple pigmentation (e.g., West and Starkey 1915, Fritsch 1916, Transeau 1933, Lynn and Brock 1969, Hoppert et al. 2004, Holzinger et al. 2010, Newsome and van Breemen 2012); this name is traditionally used in the literature. The main goal of this study was to provide insights into the abundance of phenolic compounds in *Z. ericetorum*, as well as to provide a general ecophysiological description of the performance of this alga.

*Zygogonium ericetorum* is frequently exposed to desiccation stress (Holzinger et al. 2010) and forms conspicuous macroscopic mat-like sheets that can recover physiologically upon rewetting (e.g., Hoppert et al. 2004). Cells from the top layer of these sheets may provide an additional protective function for cells in lower layers. These cells possess thick, rigid cell walls that protect against high PAR and UV-irradiation by self-shading, as described for other filamentous conjugating green algae, such as members of *Zygnema* (Harrison and Smith 2009, Holzinger et al. 2009, Pichrtová et al. 2013). Macroalgal canopies of the chlorophyte *Ulva* sp. form multiple-layered sheet-like structures in the upper littoral zone when the blades are exposed. The top layer of this sheet usually bleaches due to strong insolation, desiccation, and other abiotic stresses, thereby providing photoprotection and moisture for the sub-canopy thalli (Bischof et al. 2002). A similar self-protecting strategy seems to be widespread among the filamentous algae.

Probably the most striking visual aspect of *Z. ericetorum* is the abundance of purple pigmentation stored in the vacuoles. The earliest report, by Lagerheim (1895), described the nature of the purple cell sap as “phycoporphyrin.” Alston (1958) analyzed the pigment in more detail and proposed that the compound is an “iron-tannin”; this compound was also formed when gallic acid was mixed with the bog water collected from the algae's habitat. Most recently, the purple pigment was suggested to be a highly branched polymer of glucose, containing traces of ester-linked polyphenolic moieties such as gallic acid, which exhibits a purple color when complexed by ferric iron (Newsome and van Breemen 2012).

Galloyl glucose derivatives have been identified in the filamentous green alga *Spirogyra varians* (Zygnematales) (Nishizawa et al. 1985, Cannell et al. 1988) and their synthesis increased after cold stress (Han et al. 2009). A galloylglucopyranose lends a brownish color to the vacuoles of the freshwater ice alga *Mesotaenium berggrenii* (Remias et al. 2012b). *Mesotaenium berggreni* is also a member of the Zygnematales, which raises the possibility that this derived group of streptophycean algae has evolved the potential to synthesize certain phenols. These compounds are well known in higher plants (e.g., flavonoids) and have been proposed to serve as photoprotectants against PAR and UVR, antioxidants, repellents against grazers, and organic osmolytes that preserve intracellular activities during freezing (Remias et al. 2012a,b). Among algae, only marine Phaeophyceae are known to synthesize large amounts of tannins; these compounds are suggested to serve multiple functions including cell-wall strengthening, feeding deterrents, antimicrobial agents, and UV sunscreens (Targett and Arnold 1998, Swanson and Druehl 2002, Schönwälder 2008, Holzinger et al. 2011a). Mycosporine-like amino acids (MAAs) are common photoprotective sunscreens in many, but not all algae taxa. These biomolecules have a high absorptivity for UV-A and UV-B radiation, but are not found in most green algae except in the aeroterrestrial representatives of the Trebouxiophyceae (Karsten et al. 2005, 2007).

The ultrastructure of *Z. ericetorum* during desiccation was evaluated by Holzinger et al. (2010). The present study compared green and purple morphs of *Z. ericetorum* with respect to pigmentation and light-dependent photosynthesis, as well as to the features of their water-soluble secondary compounds, using spectrophotometric assays, high-performance liquid chromatography (HPLC), and mass-spectrometry (LC-MS). Our main goal was to evaluate the levels of the secondary photoprotective metabolites in the purple and green morphs, and to test whether elevated levels of these compounds were correlated with a higher tolerance to enhanced solar irradiation.

## Material and Methods

### Habitat and algal material

Purple and green morphotypes of *Z. ericetorum* Kützing (designated “purple morph” and “green morph”) were collected during the summers of 2009, 2010, and 2012 in a streamlet on Mt. Schönwieskopf (46°20′.862 N, 11°00′.957 E) near Obergurgl, Tyrol, Austria; the collection site at 2350 m a.s.l. was described by Holzinger et al. (2010). Climate and microclimate data for Mt. Schönwieskopf were given by Karsten et al. (2010). UVR levels (UV-A, UV-B) and PAR at the site were measured with a PMA2100 radiometer (Solar Light, Glenside, PA, USA) using sensors for PAR (PMA2132) and UV-A and UV-B (PMA 2111 and 2102). Electrical conductivity and pH of the streamlet water were obtained with a WTW Cond 330i meter (WTW Instruments, Weilheim, Germany).

For HPLC analyses, samples were collected on July 19, 2009 and July 9, 2012. Filaments of the co-occurring cyanobacterium *Scytonema* sp. were removed mechanically under a stereo microscope. For analytical purposes, the cells were washed twice with distilled water, and then ∼100 mL of the algal suspension was filtered onto glass-fiber filters (GF/C, 47 mm diameter, Whatman, Kent, UK) by vacuum-filtration (99 kPa) with a Sterifil® aseptic system (Millipore, Billerica, MA, USA). The filters with algae were immediately frozen in liquid nitrogen and lyophilized for 48 h to total dryness.

### Light and transmission electron microscopy

Light and transmission electron microscopy (TEM) were performed as previously described (Holzinger et al. 2011b, Pichrtová et al. 2013). Purple and green morphs of *Z. ericetorum* were investigated with a Zeiss 200 M (Carl Zeiss AG, Oberkochen, Germany) inverted microscope, equipped with a 63× Neofluor 1.4 NA objective lens, either in bright field or under DIC (differential interference contrast) optics. Images were captured with a Zeiss Axiocam MRc5 camera. For TEM, samples were exposed to 150 mM sucrose for a maximum period of 1 min prior to high-pressure freezing in a Leica high-pressure-freezing device (Leica, Wetzlar, Germany). Freeze substitution was carried out in 1% OsO_4_, 0.05% uranyl nitrate in acetone at −80°C for 52.5 h. The temperature was raised to −30°C within a period of 5 h and substitution was continued for 4 h at −30°C; following this treatment, the temperature was raised to 10°C within 3.5 h and maintained at that temperature for an additional 10 h before the sample was embedded in low-viscosity embedding resin (Agar Scientific, Essex, UK). Ultrathin sections were prepared with a Leica Ultramicrotome (Leica), counterstained with uranyl acetate and Reynold's lead citrate, and observed with a Zeiss LIBRA 120 TEM at 80 kV. A Proscan 2 k SSCCD camera (Proscan Electronic Systems, Lagerlechfeld, Germany) was used for image generation. Images were processed with Adobe Photoshop 7.0 software (Adobe Systems Inc., San José, CA, USA).

### Pigment extraction

The freeze-dried cells were homogenized in a Mikro-Dismembrator S mill (Sartorius, Göttingen, Germany) with a 10-mm agate-stone grinding ball in 5-mL Teflon jars, which were cooled for 10 min in liquid nitrogen prior to use. The powdered material was extracted with 4 mL of methyl-*tert*-butylether (MTBE; Sigma-Aldrich, St. Louis, MO, USA) containing 0.1% butylated hydroxytoluene (BHT; Sigma-Aldrich) to prevent oxidation of pigments. For enhanced extraction, the extract was placed in an ultrasonic bath for 15 min at room temperature and then diluted 1:1 with 20% methanol (v/v; Roth, Karlsruhe, Germany). The suspension was vortexed and cooled in a freezer overnight at −20°C to extract the sample completely. The extract was then centrifuged (1,000*g*, 5 min), which resulted in the separation of two phases, a lipophilic supernatant (MTBE) and a hydrophilic lower layer (20% methanol).

### HPLC and LC-MS

The lipophilic and hydrophilic extracts were analyzed separately by HPLC, using different methods. The lipophilic, apolar MTBE-phase (primary pigments) was vaporized using a rotary evaporator, suspended in *N*,*N*-dimethylformamide (DMF; Scharlau, Sentmenat, Spain), centrifuged at 13,000*g,* and the supernatant filtered through a polytetrafluoroethylene PTFE-filter (Millipore). The filtrate was diluted 2:1 with 50% methanol (v/v), which prepared the samples for HPLC analysis of apolar, primary pigments. The HPLC analysis of primary pigments was performed according to Remias and Lütz (2007) using an Agilent ChemStation 1100 with a binary pump and diode array detector (Agilent Technologies, Böblingen, Germany), with a binary solvent gradient using a LiChroSpher column (RP C18 5 μm 250 × 4.6 mm, Agilent Technologies). The detection wavelength was 440 nm and the peaks were identified based on retention times (RT) and absorption spectra of the peaks relative to available pigment standards (DHI LAB Products, Centralen, Denmark).

The hydrophilic polar phase (secondary pigments) was prepared for HPLC injection after centrifugation (13,000*g*, 10 min) followed by filtration through 0.45 μm regenerated cellulose syringe filters (Phenomenex, Aschaffenburg, Germany). Components of the polar phase were resolved by the same HPLC system as described above using a Phenomenex Synergi Hydro-column (RP18, 150 × 2.0 mm, 4 μm) at 25°C with a flow rate of 0.3 mL · min^−1^ and an injection volume of 25–100 μL, depending on the concentration of the extract (according to Pichrtová et al. 2013). Mobile phases: A, water + 0.5% formic acid (v/v); B, methanol + 0.5% formic acid (v/v); detection wavelengths, 280 and 350 nm. The binary linear solvent gradient started at 0% B, increased to 100% B after 40 min, followed by an 8-min post run with 100% A. Typical peaks had RTs between 5 and 25 min. A detection wavelength of 280 nm was used for semi-quantitative peak integration, to determine the relative phenol content per sample. The level of phenolics was calculated as the total peak areas normalized to the algal dry weight. The molecular masses of the most prominent secondary pigment peaks were determined by LC-MS/ESI (electrospray ionization, Esquire 3000plus, Bruker Daltonics, Bremen, Germany); for details see Remias et al. (2012b).

The purple morph was also tested for the presence of MAAs according to the improved HPLC protocol of Karsten et al. (2009), using a Phenomenex Synergi Fusion-column (RP 18, 4 μm, 250 × 3.0 mm I.D.) protected with an RP-18 guard cartridge (20 × 4 mm I.D.) of the same material. The mobile phase was 2.5% aqueous methanol (v/v) plus 0.1% acetic acid (v/v) in water, run isocratically on the Agilent ChemStation 1100 at a flow rate of 0.7 mL · min^−1^. MAAs were detected online with a photodiode array detector at 330 nm, and absorption spectra (290–400 nm) were recorded each second directly on HPLC-separated peaks.

### Spectrophotometric quantification of phenolics and tannins

The phenolic compounds of the extracts were spectrophotometrically quantified with the Folin-Ciocalteu (FC) assay, according to Makkar et al. (1993). The hydrophilic extracts (from green and purple morphs) were diluted 1:10 with *aqua bidest,* and 0.25 mL of these samples was mixed with 0.125 mL of FC-Reagent (Sigma-Aldrich, 1 M). After an incubation time of 5 min, 0.625 mL of an aqueous 20% Na_2_CO_3_ solution (w/v) was added. After a 40-min incubation period at room temperature, the absorption was measured at 725 nm (*n* = 5).

Further tests for quantification of tannins were performed with polyvinylpolypyrrolidone (PVPP), used to assay tannins by a gravimetric method (Makkar et al. 1993). The difference between total values of phenolic compounds before and after the PVPP treatment is an indicator of the levels of tannins in the samples. Twenty-five milligrams of PVPP (Sigma-Aldrich) was dissolved in 0.5 mL of each *Z. ericetorum* extract diluted 1:10, and incubated for 15 min at 4°C. The solution was then centrifuged (10,000*g*, 5 min) and a FC-assay was performed using the supernatant (*n* = 5).

Additionally, extracts were tested for the content of condensed tannins by the HCl-4-dimethylaminocinnamaldehyde assay (DMACA, Li et al. 1996) and a vanillin assay (Hagerman 2002). For the DMACA assay, 20 μL of purple or green extract of *Z. ericetorum* was mixed with 0.73 mL of aqua bidest and added to 0.25 mL of a 0.3% DMACA-HCl solution (w/v) (Sigma-Aldrich). After a 20-min incubation period at room temperature (RT), the absorption was measured at 643 nm. For the vanillin assay, 20 μL of purple or green extract of *Z. ericetorum* was diluted with 80 μL of *aqua bidest* and mixed with 500 μL of 1% vanillin-reagent (Sigma-Aldrich) in methanol (w/v) and 500 μL of 4% HCl in methanol (v/v). After incubation for 20 min at 30°C in a shaking water bath (Julabo SW23, Seelbach, Germany), the absorption was measured at 500 nm.

The purple and green morphs were also tested for the amount of hydrolyzable tannins by the rhodanine assay (Hagerman 2002); 20 μL of the extract was diluted with 80 μL of 0.1 M H_2_SO_4_ and mixed with 150 μL of 0.67% rhodanine reagent (Sigma-Aldrich) in methanol (w/v). After 5 min, 100 μL of 0.5 M KOH was added to the reaction mix, allowed to incubate for 2.5 min and then mixed with 2.5 mL of aqua bidest. After 10 min incubation at RT, the absorption was measured at 520 nm.

Absorption shifts of polar extracts (20% methanol v/v) from the purple morph were measured at different pH values (4.0, 7.0 and 12.0) and compared with other phenolic compounds such as p-cumaric acid, flavonoids, and simple phenols such as brenzcatechine. All spectrophotometric analyses were performed with a PerkinElmer Lambda 20 photometer.

### Photosynthesis and desiccation experiments

A pulse-amplitude modulated fluorometer (PAM 2000; Heinz Walz GmbH, Effeltrich, Germany) was used to evaluate photosynthetic performance. Freshly cleaned filaments from field-collected material of the green and purple morphs of *Z. ericetorum* were concentrated on five replicate Whatman GF/F glass-fiber filters by gentle filtration (99 kPa), kept moist with drops of water, and incubated in the dark for 15 min before the algal cells were exposed to 11 different photon flux densities (PFDs), each for 2 min, ranging from 11 to 406 μmol photons · m^−2^ · s^−1^. The actinic light was provided by a red light-emitting diode (LED, λ = 650 nm) of the PAM 2000 fluorometer. The distance between the fiber optic and the filter surface was kept constant at 2 mm. After each light exposure, a saturating pulse was applied, to measure the maximum fluorescence yield F_m_ and the effective photochemical efficiency as measured by ΔF/F_m_′. The relative electron transport rate of PSII (rETR) was calculated as:


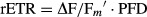


where ΔF/F_m_′, the effective PSII quantum efficiency and PFD, the photon flux density. All measurements were performed at ambient room temperature (∼22°C). Photosynthesis-irradiance (PI) curves (expressed as rETR vs. PFD) were calculated and fitted by the mathematical photosynthesis model, which either excludes photoinhibition of Webb et al. (1974) for the purple morph, or includes photoinhibition of Walsby (1997) for the green morph. From these PI curves, the light saturation point I_k_ was determined as the quotient of rETR_max_ and the initial slope α (Henley 1993).

For the desiccation experiments, freshly cleaned field-collected filaments of the green and purple morphs of *Z. ericetorum* were concentrated on five replicate Whatman GF/F glass-fiber filters by gentle filtration, and then air-dried for 150 min on a rack at ambient room temperature (∼22°C) and under 20–25 μmol photons · m^−2^ · s^−1^. After desiccation, the dried filters were rehydrated by immersion in standard growth medium, and their recovery was followed for 8 d. The optimum quantum yield (F_v_/F_m_) of photochemistry was regularly estimated during the desiccation and recovery, by PAM 2000 fluorometry. F_v_/F_m_ was determined as described by Holzinger et al. (2006).

### Statistical evaluation of the data

Results from the FC assay were analyzed by Student's *t*-test separately for the total phenols, phenols, and tannins to compare the purple and green morphs of *Z. ericetorum*. The analysis was done SPSS 18.0 for Windows (IBM Corp., Somer, NY, USA). Statistical significance of the means of the rETR as well as photosystem efficiency (Fv/Fm) of desiccated and rehydrated samples were tested with one-way ANOVA followed by a Tukey's multiple comparison test, to find subgroups of means with significant differences. Analyses were performed with InStat (GraphPad Software Inc., La Jolla, CA, USA).

## Results

### Habitat characteristics

**FIG. 1:**
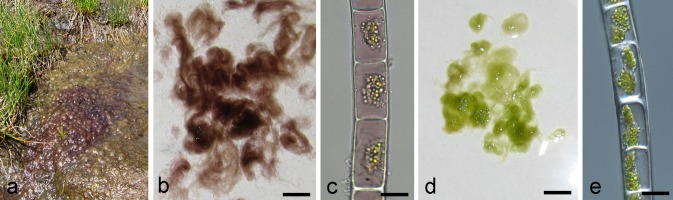
*Zygogonium ericetorum*. Habitat (a), macroscopic appearance (b and d) and photomicrographs (c and e) of; (b–c) purple morph, mostly collected in the radiation-exposed upper layer, (d–e) green morph, collected in lower layers. Scale bars, (b, d) 1 cm, (c, e) 20 μm.

*Zygogonium ericetorum* grew as macroscopic mats in an exposed ephemeral streamlet surrounded by high-alpine meadows on Mt. Schönwieskopf at 2350 m a.s.l. Two macroscopically distinguishable morphs were harvested: a thick, dark-purple, mostly sun-exposed “purple morph” in the upper layer of the mat (Fig. 1, a–c); and a greenish “green morph,” usually in the lower layer of the mat (Fig. 1, d and e). Occasionally, the green morph was also found on the sides of the streamlet, which is also exposed to sunlight. It is possible that the purple coloration is a result of cell aging.

The ambient radiation levels, measured on a sunny day, reached 2,100 μmol photons · m^−2^ · s^−1^ for PAR. The maximum UV-A and UV-B levels were 59.6 and 0.38 W · m^−2^, respectively. The water of the slowly running streamlet had electrical conductivity of 32.8 μS · cm^−1^ and pH of 6.43.

### Light microscopy

Most of the cells of *Z. ericetorum* that were collected from upper layers of the mats contained peripheral vacuoles with a purple appearance; the central cytoplasm contained the nucleus and two chloroplasts (Fig. 1c). The chloroplasts were surrounded by globular structures with an opaque appearance (Fig. 1c). In the green morph, the central chloroplasts were larger and the globular surroundings appeared homogeneous. The vacuoles at the cell periphery were colorless (Fig. 1e).

### Transmission electron microscopy

**FIG. 2:**
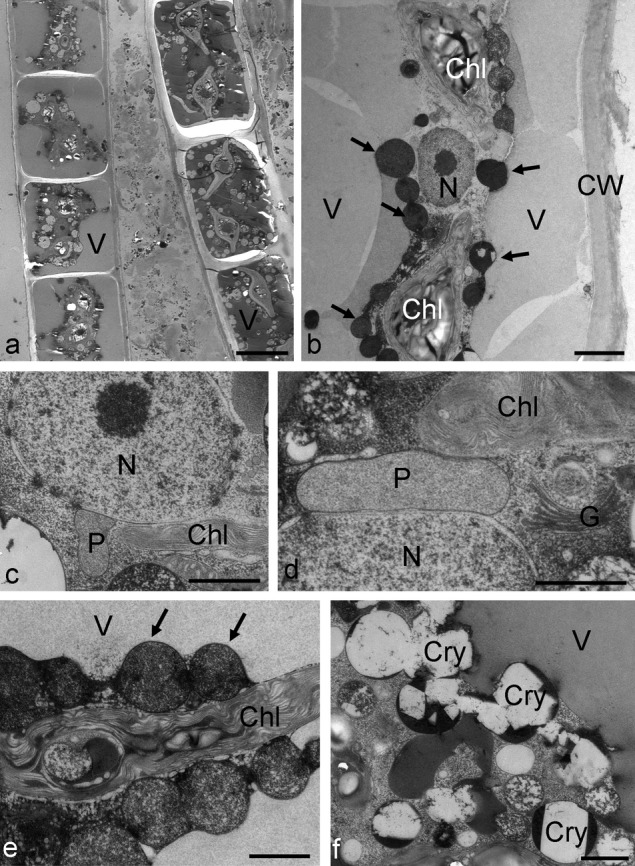
Transmission electron micrographs of the purple morph of *Zygogonium ericetorum*. (a) at low magnification, different electron densities of the filaments are visible, with a central cytoplasmic portion surrounded by large vacuoles, (b) detail of the cell center containing the nucleus, the chloroplasts are surrounded by electron-dense particles, and the cell periphery contains large vacuoles, (c) central area of the cell; the peroxisome is located directly adjacent to the nucleus, (d) individual peroxisome, nucleus, and Golgi body, (e) chloroplast is surrounded by compartments with high electron-density, (f) electron-dense compartments might contain crystals that appear electron-translucent. Abbreviations: Chl chloroplast, Cry crystal, CW cell wall, G Golgi body, N nucleus, P peroxisome, V vacuole. Scale bars, (a) 10 μm, (b) 2 μm, (c–f) 1 μm.

The features of the purple morph were significantly different from those of the green morph. TEM micrographs of high-pressure-frozen, freeze-substituted cells of the purple morph (Fig. 2) showed a small central cytoplasmic portion surrounded by large, prominent vacuoles (Fig. 2, a and b). These vacuoles were filled with an electron-opaque content (Fig. 2, a and b) and surrounded the cytoplasmic portion almost completely (Fig. 2, a and b). In individual filaments, these large vacuoles had different electron densities; Figure 2 shows a filament (left side) with electron-opaque contents and another filament (right side) with electron-dense contents (Fig. 2a). The electron opaque vacuoles always had a smooth appearance (Fig. 2b). The chloroplasts contained one pyrenoid (Fig. 2, a and b) and thylakoid membranes, and were small and pillow-shaped; occasionally smaller wings were observed protruding from the central region of the chloroplast, and these wings also contained thylakoid membranes. The nucleus was in the center of the cell, with a single peroxisome located between the nucleus and the chloroplast (Fig. 2, c and d). On the outside of the chloroplasts were electron-dense compartments with diameters from 0.5 to 2.0 μm; although most of these compartments had electron-dense contents (Fig. 2, b and e), some appeared empty. In some cases, these compartments had crystalline contents; the crystals appeared totally electron-translucent, and had likely broken out of the section (Fig. 2f). Organelles including mitochondria and Golgi bodies were also found in these areas. The cells were covered by cell walls with a diameter of up to 2 μm, and composed of several layers.

**FIG. 3:**
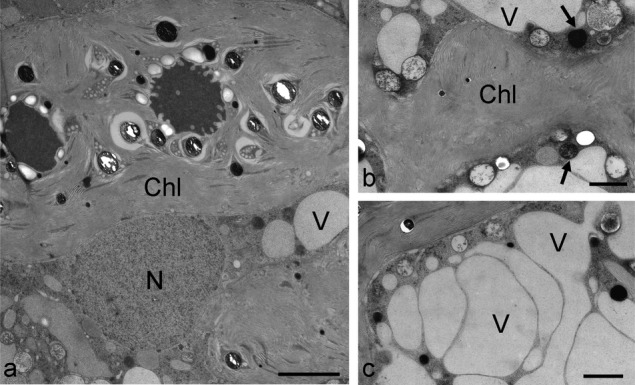
Transmission electron micrographs of the green morph of *Zygogonium ericetorum*. The chloroplasts contain pyrenoids. (a) overview of central area with nucleus and chloroplasts, (b) chloroplast is surrounded by globular structures with partially electron-dense content (arrows) and vacuoles, (c) compartmented vacuoles in the cell periphery, mostly electron-translucent. Abbreviations: Chl chloroplast, N nucleus, V vacuole. Scale bars, (a) 2 μm, (b, c) 1 μm.

In contrast, the green morph of *Z. ericetorum* had larger chloroplasts (Fig. 3a), containing electron-dense particles with a diameter of 0.5–1 μm. These structures were distinct from the plastoglobules that were occasionally observed. The chloroplasts were surrounded by smaller, highly electron-dense compartments (Fig. 3b), the vacuoles appeared compartmented, smaller than those in the purple morph, and were electron-translucent (Fig. 3c).

### Analysis of primary pigments and secondary compounds

**FIG. 4:**
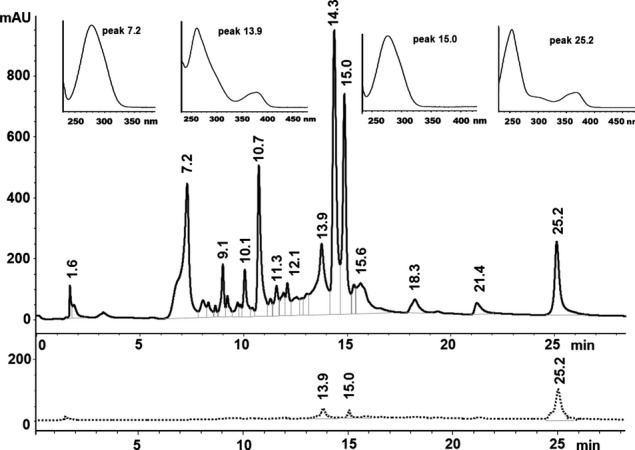
HPL-Chromatogram (280 nm) of the hydrophilic extracts (20% methanol) of the purple morph (solid line, above) and the green morph (dotted line, below) of *Zygogonium ericetorum*. The purple morph had significantly higher amounts of phenolic compounds per dry weight. All peaks had a spectral absorption maximum in the UV-B, and some of them also had a smaller maximum in the UV-A. Two compounds (peaks with RT 13.9 and 25.2 min) showed broad absorption, indicating a more complex phenolic constitution.

**FIG. 5:**
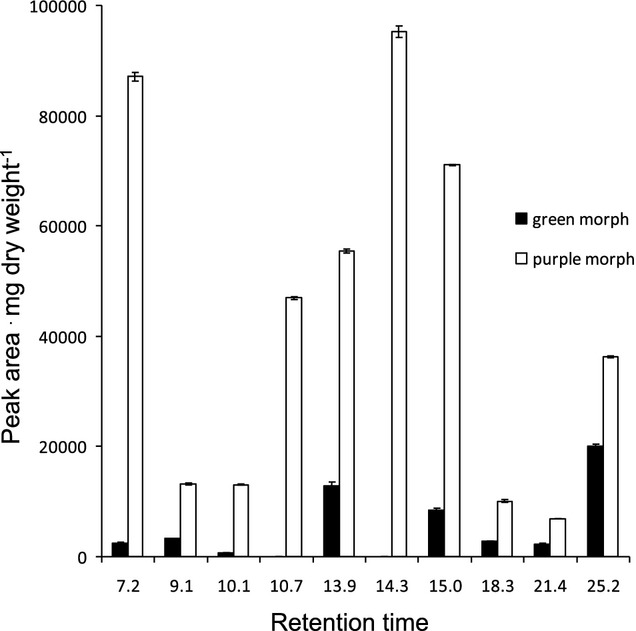
Quantification of peaks from the HPLC analysis of the secondary phenolic compounds; peak area · mg dry · weight^−1^ vs. retention time of major peaks are shown; values represent means ± SD of two HPLC runs.

The composition of primary pigments (chlorophylls and primary carotenoids) in *Z. ericetorum* was similar to that of green algae and vascular plants (data not shown). The green and purple morphs showed nearly identical chl *a*/*b* ratios, but the green morph contained about twice as much chlorophyll *a* and *b* as the purple morph. In contrast, the two morphs differed significantly in the contents of secondary metabolites, as measured by HPLC. Characteristic chromatograms of the purple and the green morph of *Z. ericetorum* are shown in Figure 4. Most soluble phenolics were found in the purple morph (at RT 7.2, 9.1, 10.1, 10.7, 13.9, 14.3, 15.0, 18.3, 21.4 and 25.2 min), and only a few occurred in more than trace amounts in the green morph (e.g., at RT 13.9, 15.0 and 25.2 min; Fig. 4). Several compounds had remarkable UV-A and UV-B-absorbencies (peaks at RT 13.9 and 25.2 min); characteristic spectra are shown for peaks at RT 7.2, 13.9, 15.0 and 25.2 min (Fig. 4). Purple *Z. ericetorum* accumulated more than four times the amount of the compounds found at RT 13.9 min, and nearly twice the amount of the compound at RT 25.2 min compared to the green morph (Fig. 5). The largest differences between the compounds eluted from the purple and green morphs were found at RT 7.2 min (35-fold more in the purple morph) and at RT 10.1 min (20-fold more in the purple morph). The amounts of most other compounds differed by between three and eight times (Fig. 5). Changes in the amounts of the purple polyphenols could not be quantified, as these compounds did not form distinct HPLC peaks.

The LC-MS analysis of the secondary metabolites, as described above, showed molecular masses of 332 u (RT 7.2 min), 646 u (RT 13.9 min), 642 u (RT 15.0 min) and 302 u (RT 25.2 min). The first compound (RT 7.2 min) showed a MSMS-fragmentation pattern typical for a pyranose moiety, as well as for a galloyl group. This compound was therefore considered to be a galloylglucopyranose. In contrast to the abundant accumulation of soluble phenolics, screening for MAAs in the extracts from purple *Z. ericetorum* did not result in any peak (data not shown).

### Spectrophotometric quantification of secondary compounds

**FIG. 6:**
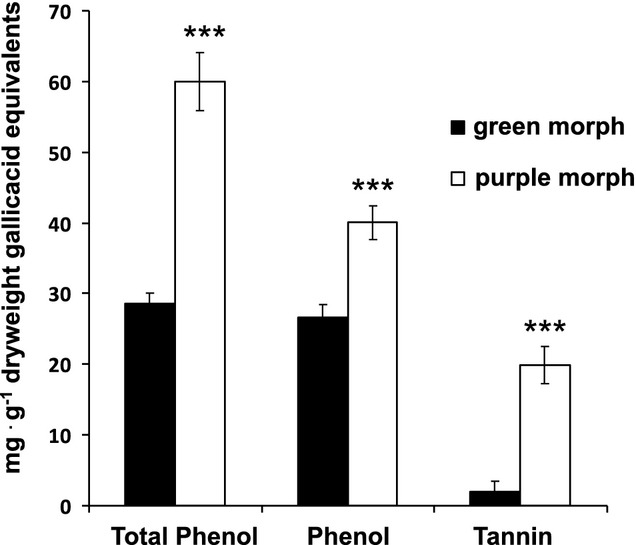
Folin-Ciocalteu (FC) assay of total phenols, phenols and tannins; comparisons of the purple and green morphs in mg · g^−1^ dry weight gallic-acid equivalents (*n* = 5, *P* < 0.001).

The FC assay indicated that the concentrations of total phenols showed a significant 2-fold increase in the purple compared to the green morph (*T*_8_ = 14.438, *P* < 0.001, Fig. 6). Phenolic compounds without tannic properties were also significantly increased (1.5-fold, *T*_8_ = 8.901, *P* < 0.001), as well as the concentration of tannins (10-fold, *T*_8_ = 11.7, *P* < 0.001, Fig. 6).

The negative results of the DMACA assay and the vanillin assay indicated that condensed tannins were not present in the extract of purple *Z. ericetorum* (data not shown). Mainly hydrolyzable tannins were detected; the rhodanine assay for the detection of hydrolyzable tannins gave positive results, which indicates that in *Z. ericetorum* mainly this type of tannic substances (glucogallin derivatives) were present.

Shifts of the absorption maxima of the polar extracts from 255 nm (pH = 4.0) and 275 nm (pH = 7.0) to 284 nm (pH = 9.0) were observed in the purple morph, which confirms the phenolic nature of the compounds (Figure S1, in the Supporting Information).

### Photosynthesis and desiccation tolerance

**FIG. 7:**
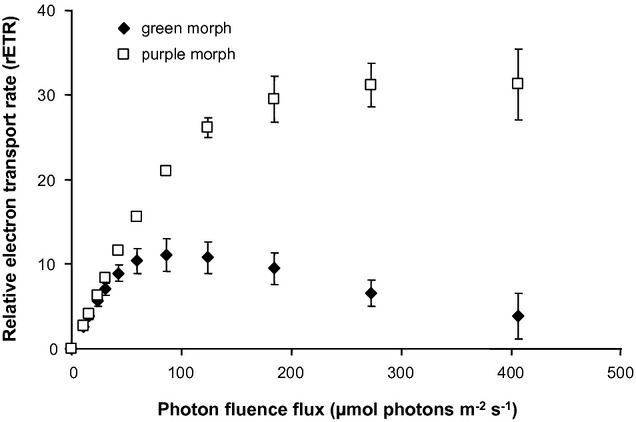
The effect of increasing photon fluence densities up to 406 μmol photons · m^−2^ · s^−1^ on the relative electron transport rate (rETR) in the green and purple morphs of *Zygogonium ericetorum* (*n* = 5, mean value ± SD).

Increasing photon fluence densities up to about 400 μmol photons · m^−2^ · s^−1^ led to conspicuously different rETR in the green and purple morphs of *Z. ericetorum* (Fig. 7). While the purple morph showed a rETR_max_ of 32.8, the green morph showed a significantly lower value of 10.9 (*P* < 0.001). Similarly, the fluorescence-based I_k_-value in the purple morph was 90.9 μmol photons · m^−2^ · s^−1^, and lower in the green one (30.2 μmol photons · m^−2^ · s^−1^; *P* < 0.001). The α-values, i.e., the positive slope at limiting photon fluence rates, were essentially identical in the two morphs: 0.361 and 0.367 μmol electrons μmol photons · m^−2^ · s^−1^ (Fig. 7). No indication of photoinhibition was observed up to the maximum photon fluence rate of 406 μmol photons · m^−2^ · s^−1^ tested in the purple morph, whereas the green morph was strongly photoinhibited (Fig. 7).

**FIG. 8:**
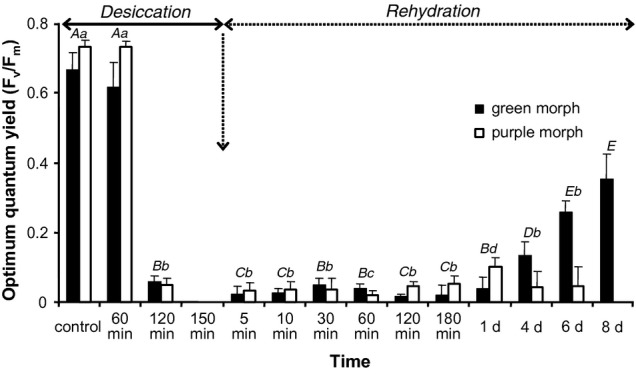
Changes in photosystem II efficiency (Fv/Fm: optimum quantum yield) in the green and purple morphs of *Zygogonium ericetorum* (*n* = 5, mean value ± SD) during 150 min desiccation, followed by 8 d recovery after rehydration with stock culture medium. Significances of differences among the treatments were calculated by one-way ANOVA (*P* < 0.001). Different letters represent significant differences among the time points as revealed by Tukey's post hoc test.

Green and purple morphs of *Z. ericetorum* were air-dried for 2.5 h at ambient room temperature, followed by rehydration and recovery over 8 d. During this desiccation experiment, the maximum PSII quantum efficiency F_v_/F_m_ was recorded at different intervals (Fig. 8). Whereas 1 h air-drying did not affect photosynthetic performance (F_v_/F_m_ values of about 0.67–0.73), after 2 h of treatment the maximum PSII quantum efficiency decreased sharply to about 5%–10% of the control level in both morphs (*P* < 0.001). Air-drying for 2.5 h completely inhibited photosynthesis (Fig. 8). After rehydration of the dried samples, only slow recovery (about 5%–14% of the control) was observed on the first day. While over the following 7 d the green morph recovered at least up to 53% of the control level (F_v_/F_m_: 0.35), the purple morph died (Fig. 8).

## Discussion

This study compared the cell structure and ultrastructure, differences in the abundance of phenolic compounds, and ecophysiological performance of purple and green morphs of *Z. ericetorum*. To our knowledge, this is the first description of the ultrastructure of *Z. ericetorum* after high-pressure freeze fixation and freeze substitution. Several unusual phenolic compounds were detected, molecular masses of several compounds were determined, and spectrophotometric tests revealed higher amounts of phenolics as well as hydrolyzable tannins in the purple morph. Measurements of the rETR demonstrated that the purple morph was substantially better protected against higher irradiation levels; however, this morph was sensitive to desiccation.

### Structure and ultrastructure

Earlier studies by Hoppert et al. (2004) and Holzinger et al. (2010) investigated the structure of field-grown *Z. ericetorum* by classical chemical fixation procedures. While the gross appearance of the cellular structure is similar after chemical fixation, some details were preserved better by the high-pressure freeze-fixation technique.

The most prominent compartments, likely containing high amounts of phenolics and tannins, were the large vacuoles surrounding the central parts of the cells. The likelihood that these compounds are present in the vacuoles is further supported by the observation that these vacuoles showed higher electron-density in the purple than in the green morph. However, the degree of electron density varied, depending on the degree of osmium tetroxide reduction. It appears particularly interesting that the freshwater ice algae *Ancylonema nordenskiöldii* (Remias et al. 2012a) and *M. berggrenii* (Remias et al. 2009) also have structurally similar vacuoles with varying electron densities. Other compartments, with particularly high-electron contrast were round and only about 1–2 μm in diameter; similar structures have been described in *Zygnema* sp. (Holzinger et al. 2009, Pichrtová et al. 2013), *M. berggrenii* (Remias et al. 2009), and *A. nordenskiöldii* (Remias et al. 2012a). These structures seem to be similar to the brown-algal phlorotannins containing physodes (Schönwälder 2008).

Remarkably, only a single peroxisome was found in the center of each cell, located between the nucleus and the chloroplast. This is particularly interesting, as a similar phenomenon was observed in other streptophycean algae of the Klebsormidiales (Honda and Hashimoto 2007, Holzinger et al. 2011b). Whether this observation has phylogenetic significance remains to be demonstrated. The electron-dense compartments covering the chloroplasts are of high ecophysiological significance. Similar structures were observed in other members of the Zygnematales, such as *Zygnema* sp., and were suggested to contain the phenolic compounds (Holzinger et al. 2010, Pichrtová et al. 2013).

### Ecological importance of phenolic compounds

As described by Holzinger et al. (2010), the top layers of the *Z. ericetorum* mats appeared especially intensely purple in the field; this coloration was lost under culture condition, in the absence of UV irradiation (unpublished observation). Since the purple morph significantly absorbs in the VIS region, the pigmentation may prevent damage from intense light to the chloroplast caused, for example, by photoinhibition or the generation of ROS. Other abiotic stresses such as desiccation and temperature fluctuations could also be responsible for increasing the content of phenolics, due to their high-antioxidative potential. While phenolics are less common in non-streptophycean green algae, in Zygnematophyceae they have been repeatedly reported (Han et al. 2007, Remias et al. 2012a,b, Pichrtová et al. 2013).

A previous study in *Z. ericetorum* showed that primary production increased when UV-A and UV-B were screened out (Cockell and Rothschild 1999). The present finding of higher amounts of UV-A and UV-B absorbing compounds (RT 13.9 and 25.2 min) in the purple morph compared to the green morph, indicates that protection against excessive irradiation is important for these algae. Interestingly, the largest increase was found in compound RT 7.2 min (molecular mass: 332 u), which was hypothesized to be galloylglucopyranose, a glucogallin (see below). Most likely, this substance plays a key role in the formation of the purple pigmentation. A recent conference contribution of Newsome and van Breemen (2012) demonstrated that the purple color of a specimen of *Z. ericetorum*, collected in Yellowstone National Park (WY, USA), was due to complexation of polyphenolic moieties, such as gallic acid, with ferric iron.

Using a different HPLC protocol (230 nm DAD-chromatogram, resin column instead of C18), Holzinger et al. (2010) found in *Z. ericetorum* two major peaks with a spectral absorption maximum at 270 nm and a shoulder at 380 nm, and another peak with absorption only at less than 330 nm and a maximum at 280 nm. Analyzing Arctic and Antarctic species of *Zygnema*, Pichrtová et al. (2013) showed four major phenolic compounds, at different RT (RT 6.5, 8.3, 16.1 and 18.8 min), as described in this study, but detected with an almost identical RP C18 method. For *M. berggrenii,* Remias et al. (2012b) reported three UV-absorbing phenolic compounds at RTs of 6.6, 17.2 and 18.1 min. Obviously, screening mechanisms exist in zygnematophycean freshwater ice algae, which probably down-regulate photosynthesis upon exposure to high irradiances, as opposed to other reported protective strategies, such as photochemical quenching and cell movement (Yallop et al. 2012); similar strategies can therefore also be expected in *Z. ericetorum*.

The absence of UV-absorbing compounds such as MAAs or secondary carotenoids suggests that mainly phenolic compounds are responsible for the photo-protection in *Z. ericetorum*. The production of phenolic compounds incurs lower metabolic costs than the production of MAAs, because phenolics do not contain nitrogen (Carreto and Carignan 2011). This is particularly important for the Zygnematales, which are mostly found in oligotrophic environments such as spring waters in the high Alps, the source of the species investigated here.

The implications of our finding of the unusual phenolic compounds in *Z. ericetorum* might be broader than simply an interaction between plants and abiotic conditions. Phenolic compounds can also discourage natural enemies of plants, including fungi, insects and mammalian herbivores (Dudt and Shurer 1994, Lattanzio et al. 2006). Although hydrolyzable tannins can inhibit the activity of alpha-glucosidase (Cannell et al. 1988), no negative effects on herbivores were revealed (Alonso et al. 2002), indicating that the higher production of phenolics and tannins may function predominantly as photo-protectants.

### Functions and sources of hydrolyzable tannins

The positive result of the FC-assay and the characteristic absorbance shifts in different alkalinities (Harborne 1998) clearly demonstrated the occurrence of phenolic compounds in the hydrophilic extracts. The positive rhodanine assay and the negative vanillin- and DMACA assays provided good evidence that the compounds detected are indeed hydrolyzable tannins.

The most likely source of hydrolyzable tannins is the shikimate pathway (SAP), due to the close similarities in chemical structure between SAP products and phenolic precursors (Ossipov et al. 2003). All genes responsible for this pathway have been identified in brown, green, and red algae and diatoms such as *Thalassiosira pseudonana* (Richards et al. 2006). Moreover, a bifunctional 3-dehydroquinate dehydratase/shikimate dehydrogenase (DHQ/SDH) was isolated in *S*. *varians*, which is phylogenetically closely related to *Z. ericetorum* (Han et al. 2009). However, besides brown algae, which contain phenolic compounds such as phlorotannins that are stored in physodes or incorporated into the cell wall (e.g., Schönwälder and Clayton 1998, Schönwälder 2008, Holzinger et al. 2011a), only a few groups of red and green algae are known to contain phenolics (e.g., Pérez-Rodríguez et al. 2001, Schmidt et al. 2012, Pichrtová et al. 2013). Similar to the function of phloroglucinol as a precursor of phlorotannins, gallic acid, glycosylated with D-glucose, acts as a precursor for hydrolyzable tannins, so-called gallotannins (Haslam 2007).

The complex mixture of phenolics and hydrolyzable tannin compounds detected in *Z. ericetorum* in the present study, and the purple polymer recently characterized by Newsome and van Breemen (2012), chemotaxonomically support the phylogenetic position of the Zygnematophyceae as a sister group to land plants (Wodniok et al. 2011). In addition, these poorly investigated phenolics could be ancestors of or substitutes for flavonoids, which have not yet been found in algae.

### Photosynthesis and desiccation tolerance

The PI curves of *Z. ericetorum* clearly indicated significant differences between the green and purple morphs in the I_k_ and ETR_max_ values, as well as in the degree of photoinhibition under moderate photon fluence rates. The ETR_max_ values for the purple morph correlate well with findings in several Antarctic and one Arctic species of *Zygnema* (Kaplan et al. 2013). In contrast, the green morph of *Z. ericetorum* showed a much lower photosynthetic performance (3-fold lower ETR_max_) and a lower I_k_-value, which point to pronounced low-light requirements, and strong photoinhibition above ∼150 μmol photons · m^−2^ · s^−1^ compared to the purple morph. The latter morph was not photoinhibited under the photon fluence rates applied, indicating much higher light requirements for photosynthesis and obviously better photoprotection. This conspicuously high light sensitivity of the green morph of *Z. ericetorum* is interesting because this alga occurs in high-alpine ephemeral streamlets (Holzinger et al. 2010). Very close (about 200 m) to the collecting site of *Z. ericetorum* in this study, biological soil crusts with the filamentous streptophycean algae *Klebsormidium crenulatum* and *K. dissectum* were collected and ecophysiologically characterized (Karsten et al. 2010, Holzinger et al. 2011b, Karsten and Holzinger 2012). Both species of *Klebsormidium* are typical aeroterrestrial taxa, and both exhibited, in contrast to the green morph of *Z. ericetorum*, high photophysiological plasticity, as reflected in the low-light requirements for photosynthesis combined with a lack of photoinhibition under enhanced irradiances. Karsten et al. (2010) argued that this high photophysiological plasticity seems to be essential for living under high-alpine conditions, because of the extreme fluctuations in abiotic factors. The steep environmental gradients include strong diurnal temperature fluctuations between day and night, occasional frost in summer, high photon fluence rates even at low temperatures, a large increase in UV-B with altitude, high impact by wind or storms resulting in drought and physical abrasion, and reduced partial pressure of carbon dioxide as an inorganic carbon source for photosynthesis. Organisms such as the aeroterrestrial *Klebsormidium* species living in alpine regions seem to be well adapted to these extreme conditions. In the case of the green morph of *Z. ericetorum,* the photophysiological properties do not support an exposed lifestyle in high-alpine streamlets, and hence the adaptive strategy seems to be to occur in shaded conditions such as underneath the purple morph. The purple morph of *Z. ericetorum* exhibited a high degree of photoprotection, which can be explained by the presence of secondary phenolic compounds.

The green and purple morphs of *Z. ericetorum* are sensitive to desiccation, and both types exhibited a sharp decrease in the optimum quantum yield after only 1–2 h air-drying. After only 2.5 h exposure to the atmosphere, the plants were rewetted and then recovered over the succeeding 8 d. Although the purple morph died, the green morph showed moderate, but very slow recovery. These observations are in strong contrast to the performance of *K. crenulatum*, which was air-dried and investigated under a similar experimental design. In this species, air-drying for 3 h also resulted in strong inhibition of the maximum PSII quantum efficiency (F_v_/F_m_), but full recovery after rehydration of the dried samples occurred after only 2 h (Karsten et al. 2010), indicating a capacity for rapid recovery and thus a rather high-desiccation tolerance. In addition to the physiological properties, the cellular ultrastructure also contributed to desiccation tolerance, as it remained more or less intact under drying conditions, and was additionally supported by flexible cross walls, resulting in a decrease in cell volume under water loss and vice versa (Holzinger et al. 2011b). Similarly, Proctor et al. (2007) reported a very rapid recovery of photosynthesis and respiration following rehydration of desiccated samples of the moss *Polytrichum formosum*. This recovery did not depend on protein synthesis and repair mechanisms, but, rather, was related to the structural integrity of the cytoskeleton. These data suggest that the reactivation of cell biological systems may have a physical component, and point to a significant role of the ultrastructure in desiccation tolerance (Holzinger et al. 2010, 2011b). In the case of *Z. ericetorum,* study of the ultrastructure of desiccated samples revealed that the vacuoles and cytoplasmatic portions appeared destroyed, whereas the nucleus and chloroplasts generally remained intact (Holzinger et al. 2010). These observations may explain why photosynthesis only partially recovered or failed to recover in the morphs of *Z. ericetorum,* after desiccation.

## Conclusions

Members of the Zygnematophyceae have been previously shown to accumulate complex phenolic substances that are uncommon in other freshwater microalgae (e.g., Han et al. 2009, Remias et al. 2012b, Pichrtová et al. 2013). The relatively high contents of phenolics and hydrolyzable tannins in the purple morph of *Z. ericetorum* are likely involved in photo-protection. Moreover, electron-dense vacuoles and electron-dense compartments were observed on the outside of the small chloroplasts in the purple morph of *Z. ericetorum* by TEM. On the one hand, screening of the phenolic compounds and hydrolyzable tannins against excessive or harmful irradiation (PAR and UV-A/B) could be the key factor in photoprotection; on the other hand, the phenolics could serve as a pool of antioxidants. Probably both processes are interacting, in such a way that increased irradiation stress may trigger increased production of phenolic substances through the shikimate pathway. Highly antioxidant active metabolites such as glucogallin are produced as first steps in the biosynthesis of complex phenolic compounds. The latter can additionally screen UV-A irradiation; however, the chemical structure of these compounds that produces the purple pigmentation could not elucidated by the methods used in this study. It is likely that harsh conditions occurring in the alpine regions stimulate microevolutionary processes, which result in the development of special physiological and morphological adaptations. The formation of algal mats could be seen as a morphological adaptation to intense light and desiccation stress, where the upper layers shade and protect algal filaments in the lower layers.

## Supporting Information

**Figure S1.** Absorption spectra between 230 and 650 nm of polar extracts (20% methanol v/v) from the purple morph of *Zygogonium ericetorum* investigated under different pH regimes (red line pH = 4.0, black line pH = 7.0, blue line pH = 12.0).
