# Supplementary figures and images for: UNUSUAL PHENOLIC COMPOUNDS CONTRIBUTE TO ECOPHYSIOLOGICAL PERFORMANCE IN THE PURPLE-COLORED GREEN ALGA ZYGOGONIUM ERICETORUM (ZYGNEMATOPHYCEAE, STREPTOPHYTA) FROM A HIGH-ALPINE HABITAT
Source: J Phycol. 2013 May 23;49(4):648–60. doi: 10.1111/jpy.12075 (PMC4370239; doi:10.1111/jpy.12075)

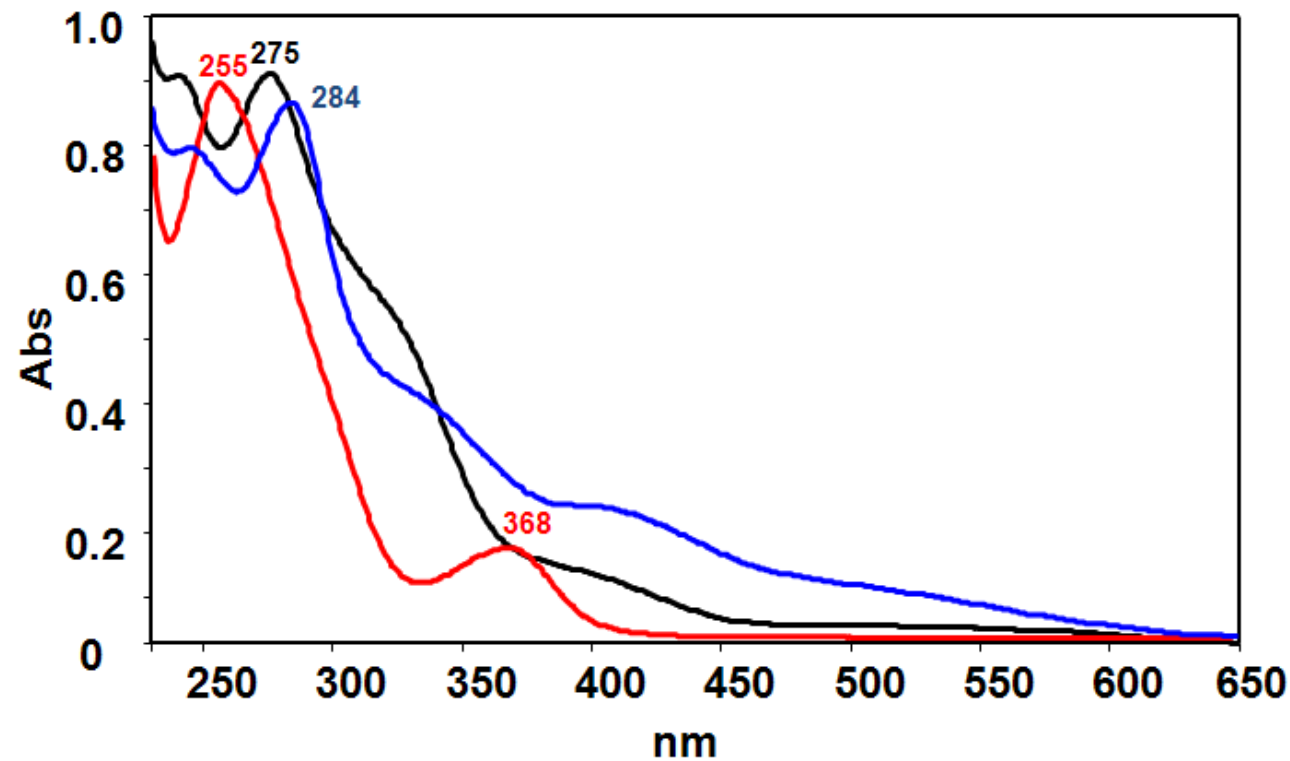

Supplement: Supplementary file 1 [file jpy0049-0648-SD1.pdf]
